# Supplementary material for: A CD4-mimetic compound enhances vaccine efficacy against stringent immunodeficiency virus challenge
Source: Nat Commun. 2018 Jun 18;9:2363. doi: 10.1038/s41467-018-04758-9 (PMC6006336; doi:10.1038/s41467-018-04758-9)
Supplement: Supplementary file 1 — Supplementary Information [file 41467_2018_4758_MOESM1_ESM.pdf]

Supplementary Information

A CD4-mimetic compound enhances vaccine efficacy against stringent immunodeficiency virus challenge

Navid Madani[1,2,3], Amy M. Princiotto[1], Linh Mach[4], Shilei Ding[5,6], Jérémie Prevost[5,6], Jonathan Richard[5,6], Bhavna Hora[7], Laura Sutherland[7], Connie A. Zhao[1], Brandon P. Conn[4], Todd Bradley[7], M. Anthony Moody[7], Bruno Melillo[8], Andrés Finzi[5,6], Barton Haynes[7], Amos B. Smith, III[8], Sampa Santra[4] and Joseph Sodroski[1,2,9]

[1]Department of Cancer Immunology and Virology, Dana-Farber Cancer Institute, Boston, MA 02215, USA

[2]Department of Microbiology and Immunobiology, Harvard Medical School, Boston, MA 02115

[3]Department of Global Health and Social Medicine, Harvard Medical School, Boston, MA 02115

[4]Center for Virology and Vaccine Research, Beth Israel Deaconess Medical Center, Harvard Medical School, Boston, MA 02215

[5]Centre de Recherche du CHUM, QC H2X 0A9, Canada

[6]Department of Microbiology, Infectious Diseases, and Immunology, Université de Montréal, Montreal, QC H2X 0A9, Canada

[7]Duke Human Vaccine Institute, Department of Medicine, Department of Immunology, Duke University Medical Center, Durham, NC 27710

[8]Department of Chemistry, University of Pennsylvania, Philadelphia, PA 19104

[9]Department of Immunology and Infectious Diseases, Harvard T.H. Chan School of Public Health, Boston, MA 02115

Supplementary Results:

Supplementary Figures 1 through 9 – pages S2 to S22

Supplementary Tables 1 through 3 – pages S23 to S25

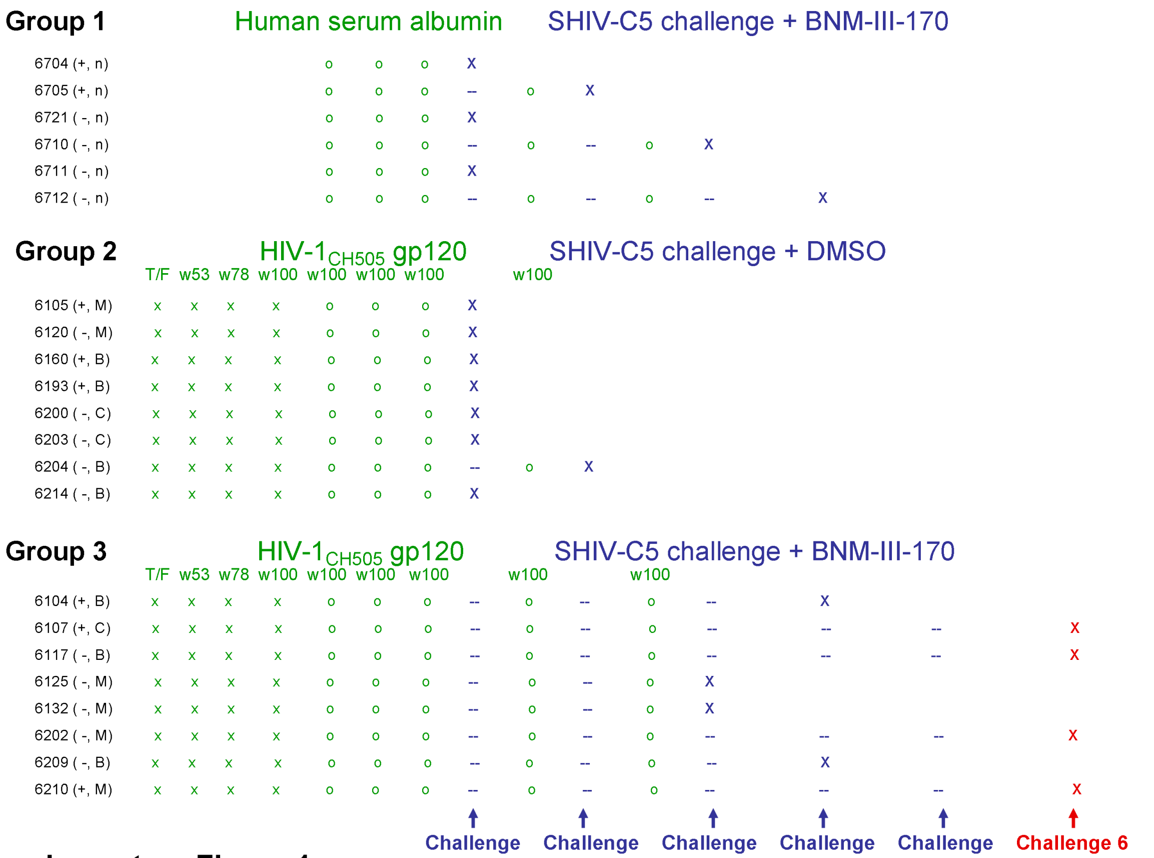

Supplementary Figure 1

Supplementary Figure 1. Study overview. The monkeys in the three study groups are listed. In parentheses are indicated the Mamu A*01 status (+ or -) of the animal, and whether the monkey was treated with a monoclonal antibody at the time of immunization (n, no treatment; M, M2850; B, Basiliximab; C, CH65 control). Monkey immunizations with either human serum albumin or HIV-1_{CH505} gp120 variants are indicated in green (x, monoclonal antibody administered following the immunization; o, no antibody treatment accompanied the immunization). The gp120 immunogens

corresponded in sequence to the transmitted/founder (T/F) virus and to Envs identified at weeks 53, 78 and 100 in the HIV-1_{CH505}-infected subject (Liao L et al., 2013. Co-evolution of a broadly neutralizing HIV-1 antibody and founder virus. Nature 496:469-476).

Challenges 1-5 of SHIV-C5 with BNM-III-170 (Groups 1 and 3) or DMSO (Group 2) are indicated in blue. Challenge 6 of the Group 3 monkeys (red) was conducted using SHIV-C5 with DMSO. The outcomes of the SHIV-C5 challenges are indicated in blue and red symbols (X, infection; --, no infection).

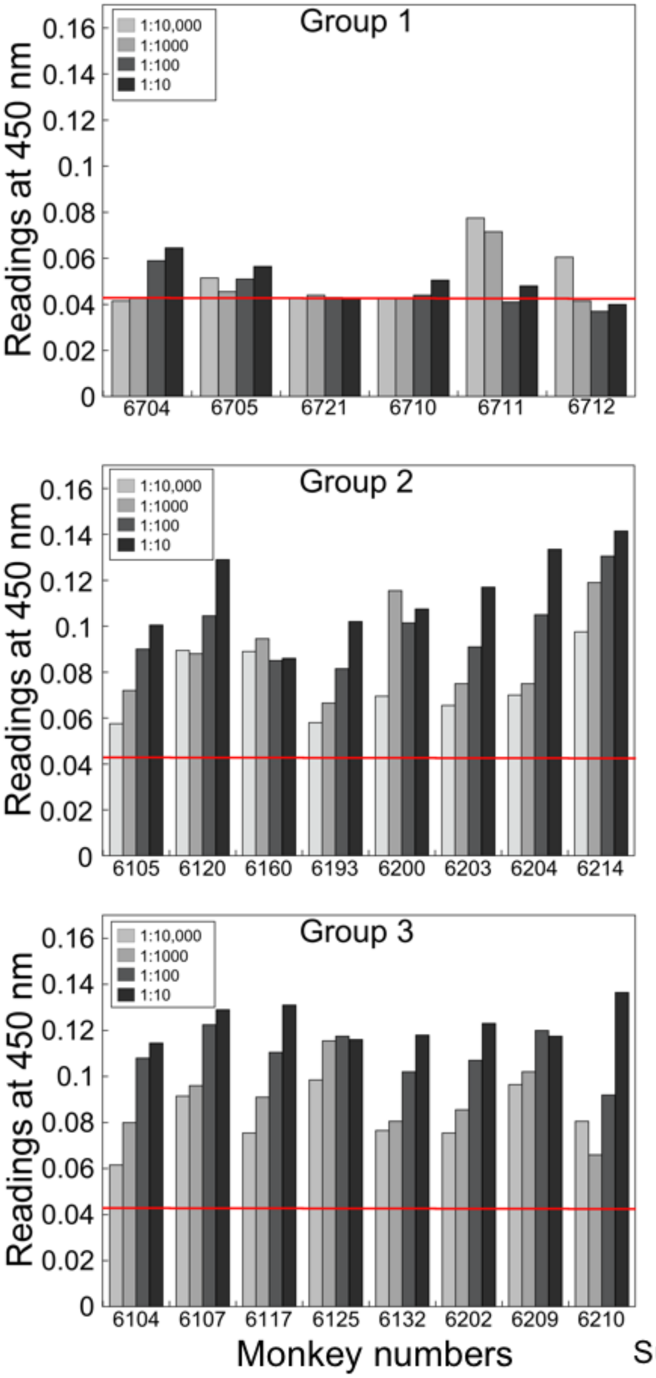

Supplementary
Figure 2

Supplementary Figure 2. Antibodies against HIV-1 gp120 Env in the sera of immunized monkeys. The antibodies against the HIV-1 gp120 Env in the sera of

immunized monkeys on the day of the first SHIV-C5 challenge were measured in an ELISA. The gp120 captured on the ELISA plate was derived from HIV-1_{YU2}, a Clade B HIV-1 strain heterologous to HIV-1_{CH505}, from which the gp120 immunogen was derived. The red line indicates the background signal, which was established by capturing human serum albumin on the ELISA plate and performing the assay without a primary antibody. As additional negative controls, the binding of uninfected monkey plasma and non-immune human sera to the gp120-coated plates was found to be equivalent to the background of the assay (data not shown). The anti-gp120 titers of the Group 2 and Group 3 monkeys both significantly differ from those of the Group 1 monkeys ($P < 0.01$, Mann-Whitney U test), but do not significantly differ from each other.

Supplementary Figure 3a

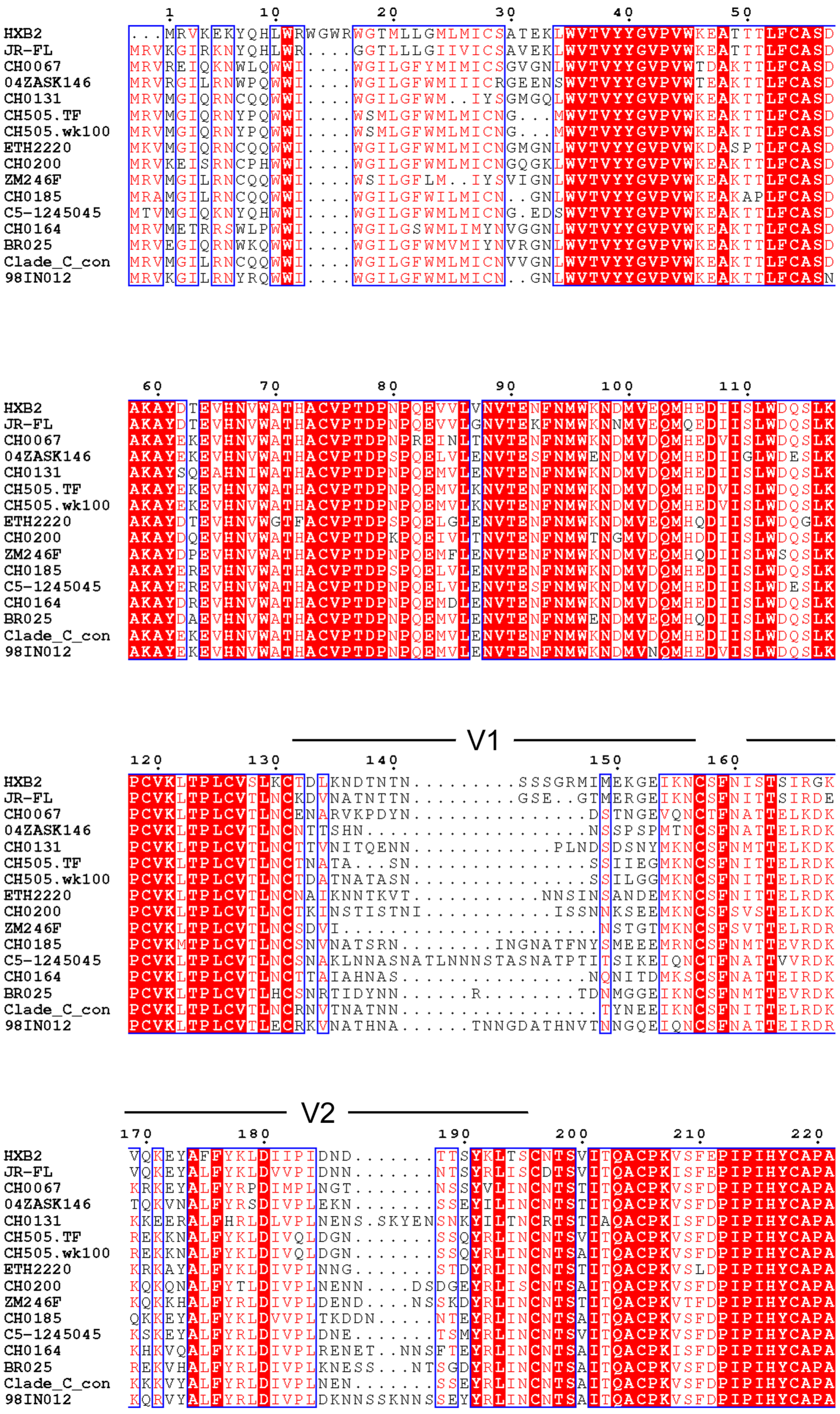

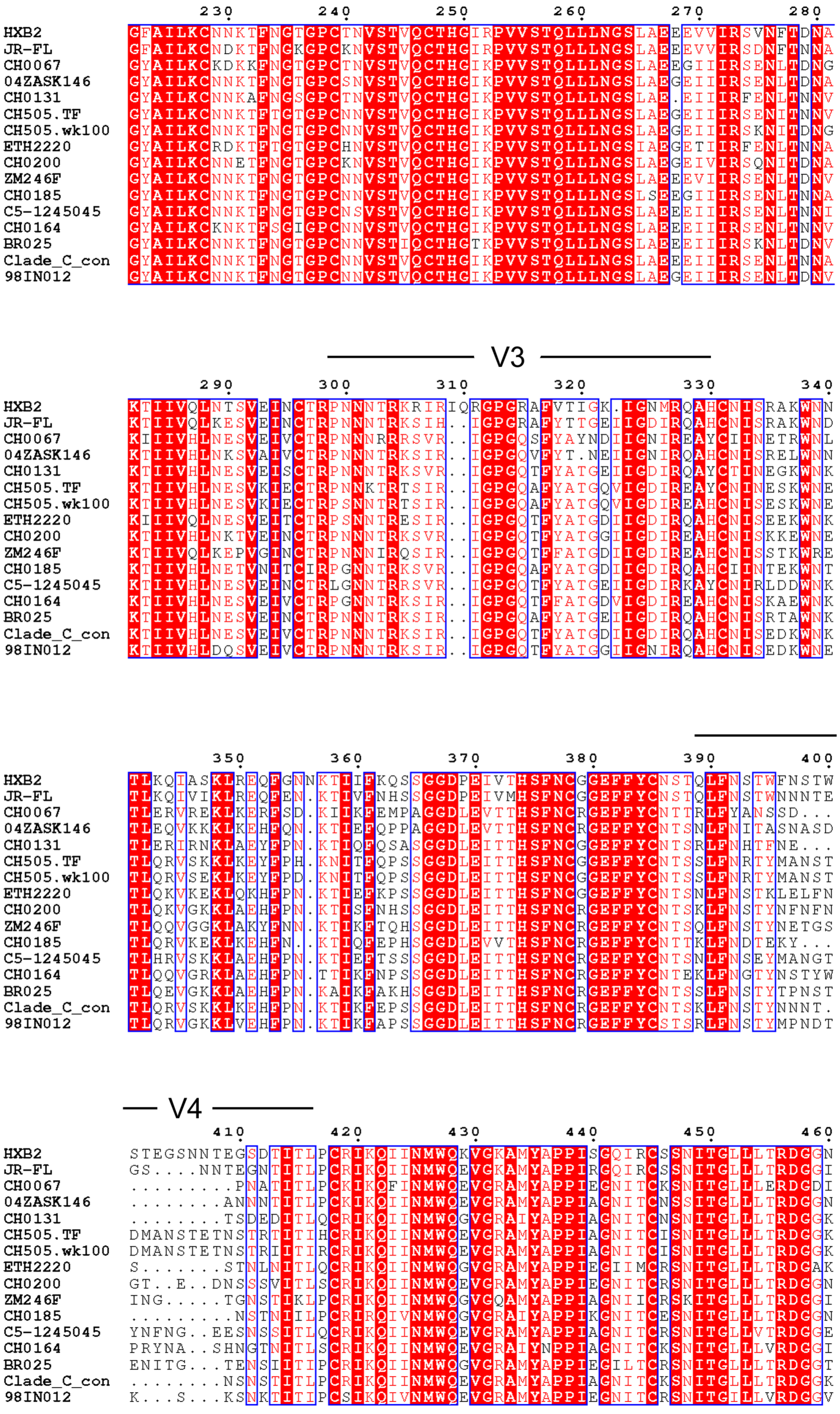

230 240 250 260 270 280
HXB2 GFAILKCNNKTFNGTGPCTNVSTVQCTHGIRPVVSTQLLLNGSLAEEEVVIRSVNFTDNA
JR-FL GFAILKCNDKTFNGKGPCKNVSTVQCTHGIRPVVSTQLLLNGSLAEEEVVIRSDNFTNNA
CH0067 GYAILKCKDKKFNGTGPCTNVSTVQCTHGIKPVVSTQLLLNGSLAEEGIIIRSENLTDNG
04ZASK146 GYAILKCNNKTFNGTGPCSNVSTVQCTHGIKPVVSTQLLLNGSLAEGEIIIRSENLTDNA
CH0131 GYAILKCNNKAFNGSGPCTNVSTVQCTHGIKPVVSTQLLLNGSLAE.EIIIRFENLTNNV
CH505.TF GYAILKCNNKTFTGTGPCNNVSTVQCTHGIKPVVSTQLLLNGSLAEGEIIIRSENITNNV
CH505.wk100 GYAILKCNNKTFNGTGPCNNVSTVQCTHGIKPVVSTQLLLNGSLAEGEIIIRSKNITDNG
ETH2220 GYAILKCRDKTFTGTGPCHNVSTVQCTHGIKPVVSTQLLLNGSIAEGETIIRFENLTNNA
CH0200 GYAILKCNNETFNGTGPCKNVSTVQCTHGIKPVVSTQLLLNGSLAEGEIVIRSQNITDNA
ZM246F GYAILKCNNKTFNGTGPCNNVSTVQCTHGIKPVVSTQLLLNGSLAEEEVIIRSENLTDNV
CH0185 GYAILKCNNKTFNGTGPCNNVSTVQCTHGIKPVVSTQLLLNGSLSEEGIIIRSENLTNNA
C5-1245045 GYAILKCNNKTFNGTGPCNSVSTVQCTHGIKPVVSTQLLLNGSLAEEEIIIRSENLTNNI
CH0164 GYAILKCKNKTFSGIGPCNNVSTVQCTHGIKPVVSTQLLLNGSLAEEEIIIRSENLTNNV
BR025 GYAILKCNNKTFNGTGPCNNVSTIQCTHGTKPVVSTQLLLNGSLAEEEIIIRSKNLTDNV
Clade_C_con GYAILKCNNKTFNGTGPCNNVSTVQCTHGIKPVVSTQLLLNGSLAEEEIIIRSENLTNNA
98IN012 GYAILKCNNKTFNGTGPCNNVSTVQCTHGIKPVVSTQLLLNGSLAEGEIIIRSENLTDNV
V3
290 300 310 320 330 340
HXB2 KTIIVQLNTSVEINCTRPNNNTRKRIRIQRGPGRAFVTIGK.IGNMRQAHCNISRAKWNN
JR-FL KTIIVQLKESVEINCTRPNNNTRKSIH..IGPGRAFYTTGEIIGDIRQAHCNISRAKWND
CH0067 KIIIVHLNESVEIVCTRPNNNRRRSVR..IGPGQSFYAYNDIIGNIREAYCIINETRWNL
04ZASK146 KTIIVHLNKSVAIVCTRPNNNTRKSIR..IGPGQVFYT.NEIIGNIRQAHCNISRELWNN
CH0131 KTIIVHLNESVEISCTRPNNNTRKSVR..IGPGQTFYATGEIIGDIRQAYCTINEGKWNK
CH505.TF KTIIVHLNESVKIECTRPNNKTRTSIR..IGPGQAFYATGQVIGDIREAYCNINESKWNE
CH505.wk100 KTIIVHLNESVKIECTRPSNNTRTSIR..IGPGQAFYATGQVIGDIREAHCNISESKWNE
ETH2220 KIIIVQLNESVEITCTRPSNNTRESIR..IGPGQTFYATGDIIGDIRQAHCNISEEKWNK
CH0200 KTIIVHLNKTVEINCTRPNNNTRKSVR..IGPGQTFYATGGIIGDIREAHCNISKKEWNE
ZM246F KTIIVQLKEPVGINCTRPNNNIRQSIR..IGPGQTFFATGDIIGDIREAHCNISSTKWRE
CH0185 KTIIVHLNETVNITCIRPGNNTRKSVR..IGPGQAFYATGDIIGDIRQAHCIINTEKWNT
C5-1245045 KTIIVHLNESVEINCTRLGNNTRRSVR..IGPGQTFYATGEIIGDIRKAYCNIRLDDWNK
CH0164 KTIIVHLNESVEIVCTRPGNNTRKSIR..IGPGQTFFATGDVIGDIREAHCNISKAEWNK
BR025 KTIIVHLNESVEINCTRPNNNTRKSIR..IGPGQAFYATGEIIGDIRQAHCNISRTAWNK
Clade_C_con KTIIVHLNESVEIVCTRPNNNTRKSIR..IGPGQTFYATGDIIGDIRQAHCNISEDKWNK
98IN012 KTIIVHLDQSVEIVCTRPNNNTRKSIR..IGPGQTFYATGGIIGNIRQAHCNISEDKWNE
350 360 370 380 390 400
HXB2 TLKQIASKLREQFGNNKTIIFKQSSGGDPEIVTHSFNCGGEFFYCNSTQLFNSTWFNSTW
JR-FL TLKQIVIKLREQFEN.KTIVFNHSSGGDPEIVMHSFNCGGEFFYCNSTQLFNSTWNNNTE
CH0067 TLERVREKLKERFSD.KIIKFEMPAGGDLEVTTHSFNCRGEFFYCNTTRLFYANSSD...
04ZASK146 TLEQVKKKLKEHFQN.KTIEFQPPAGGDLEVTTHSFNCRGEFFYCNTSNLFNITASNASD
CH0131 TLERIRNKLAEYFPN.KTIQFQSASGGDLEITTHSFNCGGEFFYCNTSRLFNHTFNE...
CH505.TF TLQRVSKKLKEYFPH.KNITFQPSSGGDLEITTHSFNCGGEFFYCNTSSLFNRTYMANST
CH505.wk100 TLQRVSEKLKEYFPD.KNITFQPSSGGDLEITTHSFNCGGEFFYCNTSSLFNRTYMANST
ETH2220 TLQKVKEKLQKHFPN.KTIEFKPSSGGDLEITTHSFNCGGEFFYCNTSNLFNSTKLELFN
CH0200 TLQKVGKKLAEHFPN.KTISFNHSSGGDLEITTHSFNCRGEFFYCNTSKLFNSTYNFNFN
ZM246F TLQQVGGKLAKYFNN.KTIKFTQHSGGDLEITTHSFNCRGEFFYCNTSQLFNSTYNETGS
CH0185 TLQRVKEKLKEHFN..KTIQFEPHSGGDLEVVTHSFNCRGEFFYCNTTKLFNDTEKY...
C5-1245045 TLHRVSKKLAEHFPN.KTIEFTSSSGGDLEITTHSFNCRGEFFYCNTSNLFNSEYMANGT
CH0164 TLQQVGRKLAEHFPN.TTIKFNPSSGGDLEITTHSFNCRGEFFYCNTEKLFNGTYNSTYW
BR025 TLQEVGKKLAEHFPN.KAIKFAKHSGGDLEITTHSFNCRGEFFYCNTSSLFNSTYTPNST
Clade_C_con TLQRVSKKLKEHFPN.KTIKFEPSSGGDLEITTHSFNCRGEFFYCNTSKLFNSTYNNNT.
98IN012 TLQRVGKKLVEHFPN.KTIKFAPSSGGDLEITTHSFNCRGEFFYCSTSRLFNSTYMPNDT
V4
410 420 430 440 450 460
HXB2 STEGSNNTEGSDTITLPCRIKQIINMWQKVGKAMYAPPISGQIRCSSNITGLLLTRDGGN
JR-FL GS....NNTEGNTITLPCRIKQIINMWQEVGKAMYAPPIRGQIRCSSNITGLLLTRDGGI
CH0067 .........PNATITLPCKIKQFINMWQEVGRAMYAPPIEGNITCKSNITGLLLERDGDI
04ZASK146 .........ANNTITLPCKIKQIINMWQEVGRAMYAPPIAGNITCNSSITGLLLTRDGGN
CH0131 .........TSDEDITLQCRIKQIINMWQGVGRAIYAPPIAGNITCNSNITGLLLTRDGGK
CH505.TF DMANSTETNSTRTITIHCRIKQIINMWQEVGRAMYAPPIAGNITCISNITGLLLTRDGGK
CH505.wk100 DMANSTETNSTRIITIRCRIKQIINMWQEVGRAMYAPPIAGNITCISNITGLLLTRDGGK
ETH2220 S.......STNLNITLQCRIKQIINMWQGVGRAMYAPPIEGIIMCRSNITGLLLTRDGAK
CH0200 GT..E..DNSSSVITLSCRIKQIINMWQEVGRAMYAPPIEGNITCRSNITGLLLTRDGGN
ZM246F ING.....TGNSTIKLPCRIKQIINMWQGVGQAMYAPPIAGNIICRSKITGLLLTRDGGI
CH0185 .........NSTNIILPCRIRQIVNMWQGVGRAIYAPPIKGNITCESNITGLLLTRDGGN
C5-1245045 YNFNG..EESNSSITLQCRIKQIINMWQEVGRAMYAPPIAGNITCRSNITGLLVTRDGGE
CH0164 PRYNA..SHNGTNITLSCRIKQIINMWQEVGRAIYNPPIAGNITCKSNITGLLLVRDGGI
BR025 ENITG...TENSIITIPCRIKQIINMWQGVGRAMYAPPIEGILTCRSNITGLLLTRDGGT
Clade_C_con .........NSNSTITLPCRIKQIINMWQEVGRAMYAPPIAGNITCKSNITGLLLTRDGGK
98IN012 K...S...KSNKTITIPCSIKQIVNMWQEVGRAMYAPPIEGNITCRSNITGILLVRDGGV

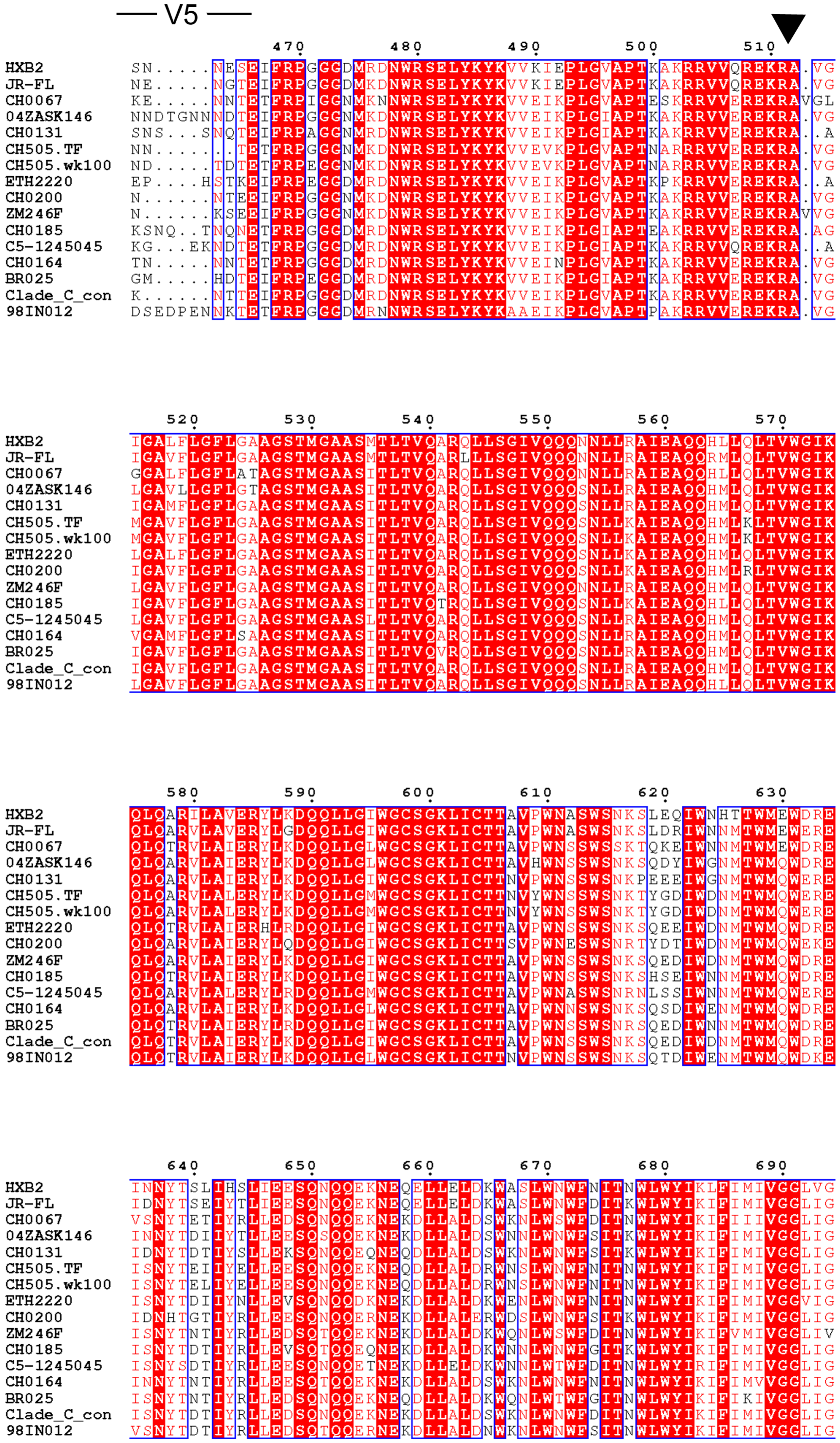
V5
470
480
490
500
510
520
530
540
550
560
570
580
590
600
610
620
630
640
650
660
670
680
690
HXB2
JR-FL
CH0067
04ZASK146
CH0131
CH505.TF
CH505.wk100
ETH2220
CH0200
ZM246F
CH0185
C5-1245045
CH0164
BR025
Clade_C_con
98IN012

```
                     700       710       720        730       740       750
HXB2         LRIVFAVLSIVNRVRQGYSPLSFQTHLPTP.RGPDRPEGIEEEGGERDRDRSIRLVNGSL
JR-FL        LRIVFTVLSIVNRVRQGYSPLSFQTLLPAP.RGPDRPEGIEEEGGERDRDRSGRLVNGFL
CH0067       LRIIFGVLTIVRRVRQGYSPLSFQTLIPNP.RGLDRLGRIEEEGGEQDKDRSIRLVNGFL
04ZASK146    LRIILGVLSIVKRVRQGYSPLSFQTLPPNP.RGPDRLRGIEEEGGEQDKDRSIRLVSGFL
CH0131       LRIIFAVLSIVNRVRQGYSPLSFQTLIPNP.RGLDRLERIEEEGGEQDRDRSIRLVNGFL
CH505.TF     LRIIFAVLSLVNRVRQGYSPLSFQTLIPSP.RGPDRPGGIEEEGGEQDRNRSTRLVSGFL
CH505.wk100  LRIIFAVLSLVNRVRQGYSPLSLQTLIPSP.RGPDRPGGIEEEGGEQDRNRSTRLVSGFL
ETH2220      LRIIFAVLSIVNRVRQGYSPLSFQTLIPHP.RGPDRLGGIEEEGGEQGRDRSIRLVNGFL
CH0200       LRIIFAVLSIVNRVRQGYSPLSFQTLIPNP.RGLDRLGRIEEEGGEQDKDRSIRLVNGFL
ZM246F       LRIIFAVFSIVNRVRQGYSPLSFQTLTPAQ.RGPDRLGRIEEEGGEQDRVRSVRLVSGFL
CH0185       LRIIFAVLSIVNRVRQGYSPLSFQTLTPNP.RGPDRLGGIEEEGGEQDKGRSIRLVNGFL
C5-1245045   LRIIFAVLSIVNRVRQGYSPLSFQTLFPNP.REPDRLGGIEEEGGEQDRNRSIRLVSGFL
CH0164       LRIIFAVLSIVNRVRQGYSPLSFQTLTPNPTRGPDRLGGIEEEGGEQDRGRSIRLVSGLL
BR025        LRIIFAVLSIVNRVRQGYSPLSFQTLTPNP.RGPDRLGGIEEEGGEQDRDRSIRLVSGFL
Clade_C_con  LRIIFAVLSIVNRVRQGYSPLSFQTLTPNP.RGPDRLGRIEEEGGEQDRDRSIRLVSGFL
98IN012      LRIIFAVLSIVNRVRQGYSPLSFQTLTPNP.RGPDRLGRIEEEGGGQDRDRSIRLVNGFL

                      760       770       780              790       800
HXB2         ALIWDDLRSLCLFSYHRLRDLLLIVTRIVELLGR.......RGWEALKYWWNLLQYWSQE
JR-FL        ALIWVDLRSLCLFSYHRLRDLLLTVTRIVELLGR.......RGWEVLKYWWNLLQYWSQE
CH0067       ALAWDDLRSLCLFSYRQLRDFILIVARAVELLGRSSLRGLQRGWEVLKYLGNLVQYWGLE
04ZASK146    ALVWEDLRSLCLFSYHRLRDLILIAGRAAELLGRSSLRGLQTGWQALKYLGSLVQYWGLE
CH0131       ALAWDDLRSLCLFSYHRLRDCVLIVTRVVELLGH......QRGWEILKYLGSLAQYWGLE
CH505.TF     ALVWDDLRSLCLFIYHRLRDFILIAARAGELLGRSSLKGLRRGWEALKYLGSLVQYWGLE
CH505.wk100  ALAWDDLRSLCLFIYHRLRDFILIAARAGELLGRSSLKGLRRGWEALKYLGSLVQYWGLE
ETH2220      AIFWDDLRSLCLFSYHRLRDLILIAARTVELLGRSSLKGLQRGWETLKYLGSLVQYWGLE
CH0200       ALFWDDLRNLCLFSYHQLRDLTLVTARAVELLGSSSLRGIQRGWEVLKYLGSLVQYWCLE
ZM246F       ALAWDDLRSLCLFCYRQLRDLLLIATRAAELLGRSSLRGLQRGWETLKYLGNLVQYWVLE
CH0185       ALVWDDLRSLCLFSYHQLRDFILIVTRAVELLGRSSLKGLQRGWEALKYLGSLVQYWGLE
C5-1245045   SLAWDDLRSLCLFSYHRLRDFILIVVRTVKLLGHSSLRGLQRGWETLKYLGSLVQYWGLE
CH0164       ALAWDDLRSLCLFSYHRLRDFLLVAVRVVELLGRSSLRGLQRGWEALKYLGSLVQYWGLE
BR025        ALAWDDLRSLCLFSYHRLRDLILIAARAVELLGRSSLKGLQRGWEILKYLGGLVQYWSLE
Clade_C_con  ALAWDDLRSLCLFSYHRLRDFILVAARAVELLGRSSLRGLQRGWEALKYLGSLVQYWGLE
98IN012      ALAWDDLWSLCLFSYHRLRDLILVTARAVELLGHSSLRGIQRGWEALKYLGGIVQYWGLE

                810       820       830       840       850
HXB2         LKNSAVSLLNATAIAVAEGTDRVIEVVQGACRAIRHIPRRIRQGLERILL
JR-FL        LKNSAVSLLNATAIAVAEGTDRIIEALQRTYRAILHIPTRIRQGLERALL
CH0067       LKRSAISLFDTLAITVAEGTDRILELVQRICRAIRNIPIRIRQGFEAALL
04ZASK146    LKKSAINLFDTTAIVVAEGTDRLIEGLQGIGRAIYNIPRRIRQGFEAALL
CH0131       LKQSAINLLDTIAIAVAEGTDRIIEVIQRICRAICNIPTRIRQGFEAALQ
CH505.TF     LKRSAISLLDTLAIAVGEGTDRILEFVLGICRAIRNIPTRIRQGFETALL
CH505.wk100  LKRSAISLLDTLAIAVGEGTDRILEFVLGICRAIRNIPTRIRQGFETALL
ETH2220      LKKSAINLLNTTAIVVGEGTDRFIELIQRIWRAFCNIPRRIRQGLEAALQ
CH0200       LKRSAISLLDTIAIAVAEGTDRIIELIQRICRAIYHIPRRIRQGFEAALQ
ZM246F       LKRSATSLLDTIAIAVAEGTDRIIELIQGIGRAIYNIPRRIRQGFEAALQ
CH0185       LKKSAINLLDTIAIAVAEGTDRIIEIIQAFCRGIYNIPRRIRQGFEAALL
C5-1245045   LKKSAISLLDTIAIAVAEGTDRIIEIIQRICRAILHIPTRIRQGFEAALQ
CH0164       LKKSAISLVDTIAIAVAEGTDRIIELLRIICRAILNIPTRIRQGFEAALL
BR025        LKKSAISLFDTIAIAVAEGTDRIIEVIQGIWRAICNIPRRIRQGFEAALQ
Clade_C_con  LKKSAISLLDTIAIAVAEGTDRIIELIQRICRAIRNIPRRIRQGFEAALQ
98IN012      LKKRAISLLDTVAIAVAEGTDRIIEFLQRICRAIRNIPRRIRQGFEAALQ
```

Supplementary Figure 3b

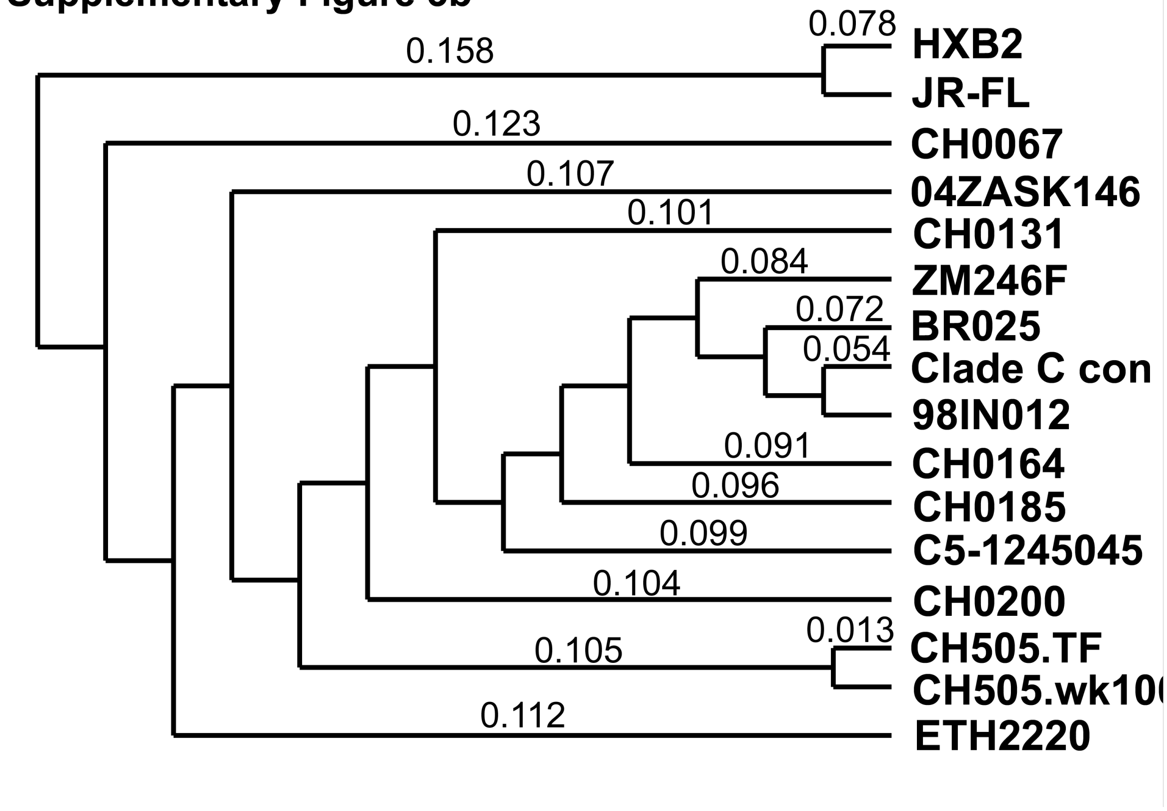

Supplementary Figure 3. Env sequence comparisons. (**a**) The Env amino acid sequences of HIV-$1_{CH505.TF}$ and HIV-$1_{CH505.wk100}$, from which the gp120 immunogens were derived, were aligned with those of HIV-1_{C5} (1245045) (in the challenge SHIV) and with Envs from reference Clade C and Clade B HIV-1 strains, using Clustal Omega (Sievers F, Wilm A, Dineen D, Gibson TJ, Karplus K, Li W, Lopez R, McWilliam H, Remmert M, Söding J, Thompson JD, Higgins DG. 2011. Fast, scalable generation of high-quality protein multiple sequence alignments using Clustal Omega. Mol Syst Biol *7*:539). A Clade C HIV-1 consensus sequence (Clade C con) was included in the analysis. The Env amino acid residues are numbered according to that of the Clade B

HIV-1_{HXB2} standard reference strain. Amino acid residues conserved in all of the HIV-1 Envs evaluated are highlighted with a red background, and regions with conservative amino acid substitutions are boxed in blue, with the major residue type in red letters. The V1-V5 variable regions of gp120 are labeled, and the site of gp120-gp41 proteolytic cleavage is indicated (black triangle). Note the relatively long V1 region of the HIV-1_{C5} Env. The Env sequence alignment was illustrated with ESPript 3 (Robert X, Gouet P. 2014. Deciphering key features in protein structures with the new ENDscript server. Nucleic Acids Res *42*: W320-4). (**b**) The Clustal Omega alignment in **a** was used to prepare a dendrogram showing the relatedness of Env protein sequences from the indicated HIV-1 strains, using the unweighted pair group method with arithmetic mean (UPGMA). The distances from the nodes to the branch tips of the dendrogram are indicated.

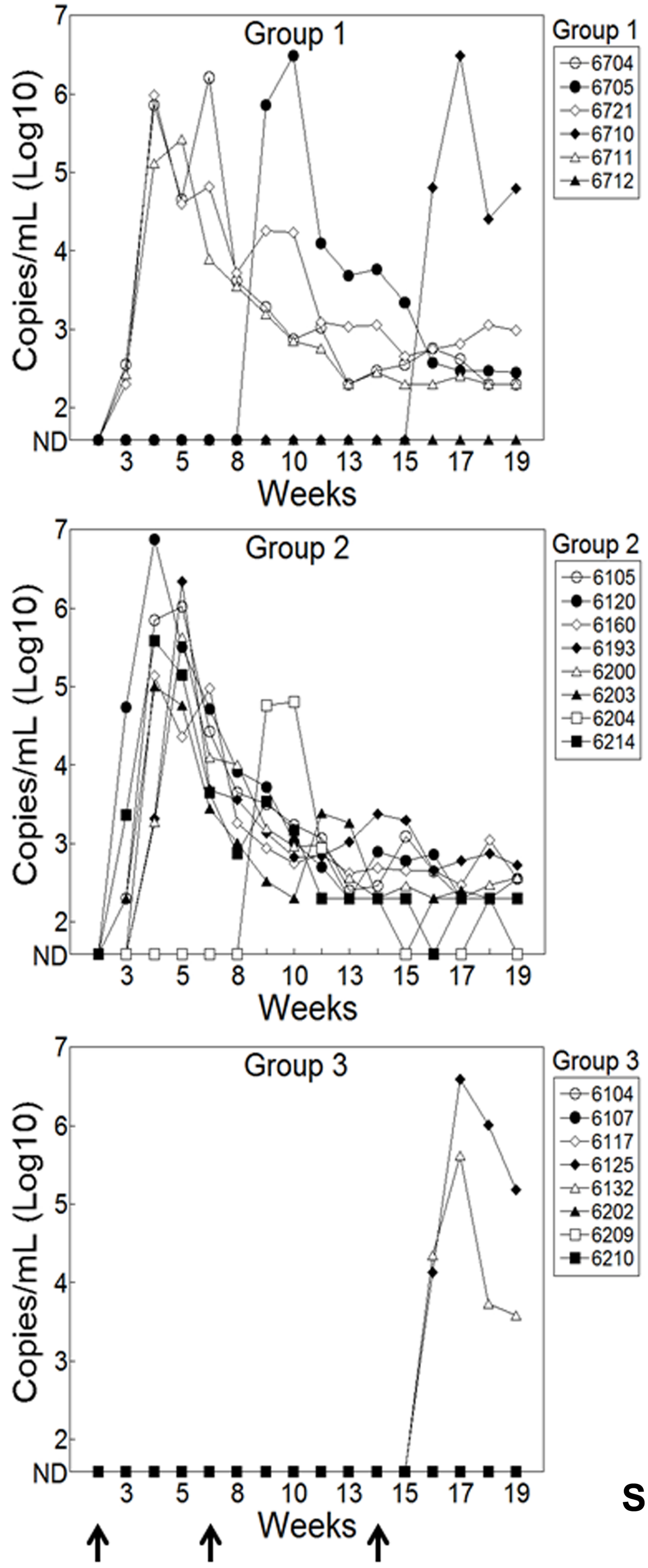

Supplementary Figure 4

Supplementary Figure 4. Plasma viremia in the SHIV-C5-challenged monkeys. The number of SHIV RNA copies per milliliter of plasma at the indicated time points after the different SHIV-C5 challenges is shown on a log scale. The arrows indicate the times of the SHIV-C5 challenges. ND, not detected.

Supplementary Figure 5

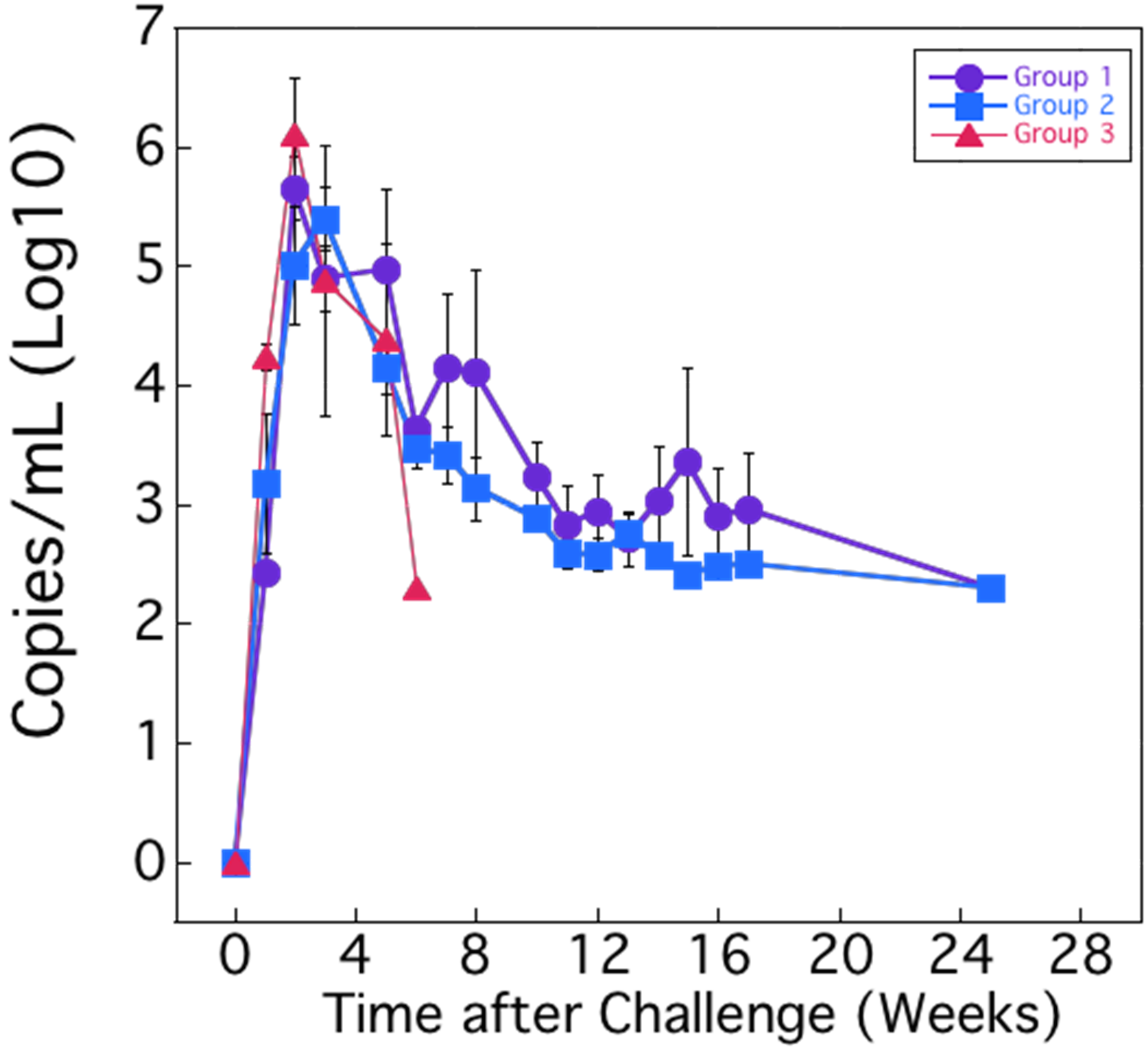

Supplementary Figure 5. Comparison of average levels of SHIV-C5 viremia in the Group 1-3 monkeys. The average levels of viremia in the Group 1, Group 2 and Group 3 monkeys following SHIV-C5 Challenges 1-3 are shown, with standard deviations.

Supplementary Figure 6a

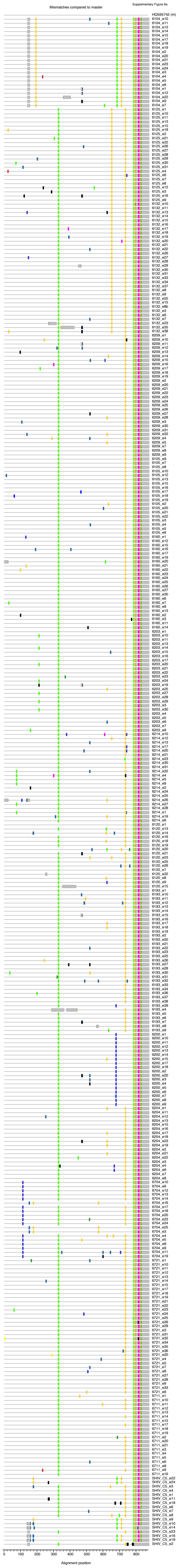

Supplementary Figure 6b

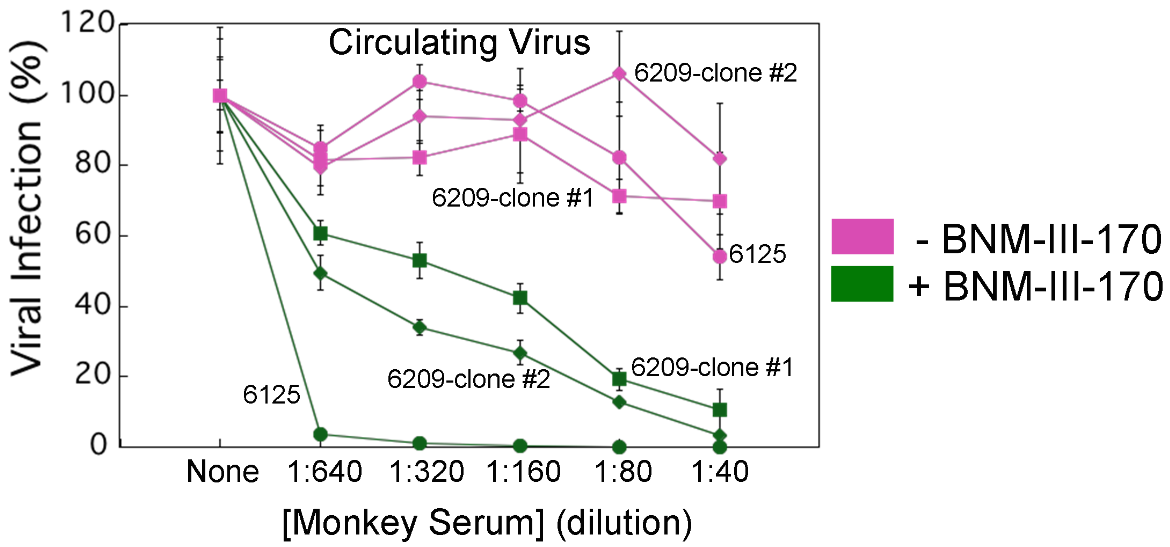

Supplementary Figure 6. Properties of Envs in viruses from infected monkeys. (**a**) A Highlighter plot depicts the predicted Env sequences of the circulating viruses from the indicated monkeys, at the time of peak viremia. Mismatches compared to the HIV-1_{C5} Env (HQ595742) are depicted. The Env sequences from the SHIV-C5 challenge stock are shown at the bottom of the figure. (**b**) The circulating viruses from the two Group 3 monkeys (6125 and 6132) that became infected after Challenge 3 exhibited the same Env amino acid sequences (see (**a**) above). We evaluated BNM-III-170 sensitization of the viruses from a Group 3 monkey (6125) that became infected after Challenge 3 and a control Group 3 monkey (6209) that remained uninfected after Challenge 3. Recombinant HIV-1 pseudotyped with the circulating virus Envs was tested for sensitivity to neutralization by serum from the infected monkey on the day of

challenge, in the presence of 20 μM BNM-III-170 or an equivalent volume of DMSO. The neutralization assays were conducted similarly to those reported in Figure 2 in the main section of the manuscript. The means and standard deviations of values obtained from triplicate assays are shown. The inhibitory concentrations (IC_{50}'s) of BNM-III-170 were 51.6+/-13 μM (C5), 11.7+/-2.4 μM (6209) and 6.0+/-0.5 μM (6125).

Supplementary Figure 7

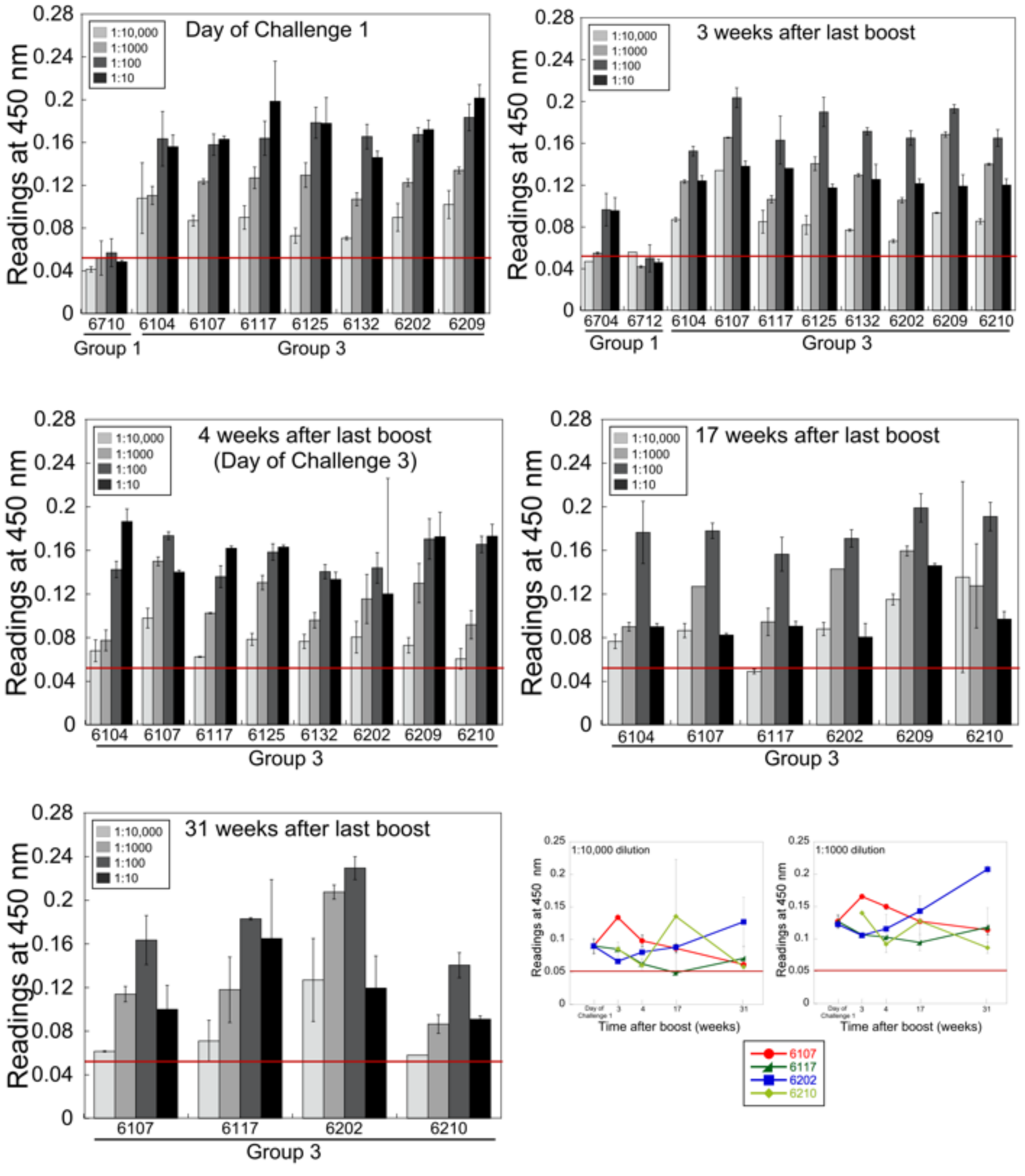

Supplementary Figure 7. Persistence of antibodies against HIV-1 gp120 in immunized monkeys. The antibodies against HIV-1 gp120 in the sera of immunized monkeys were measured in an ELISA assay, as in Supplementary Figure 2. The sera were collected from the monkeys on the day of SHIV-C5 Challenge 1 or at the indicated times after the last gp120 boost. The means and standard deviations from triplicate assays are shown. The red line indicates the background signal. In the lower right panel, the ELISA readings are shown for the 1:10,000 and 1:1000 dilutions of sera from the four uninfected monkeys in Group 3 as a function of time following the last gp120 boost.

Supplementary Figure 8

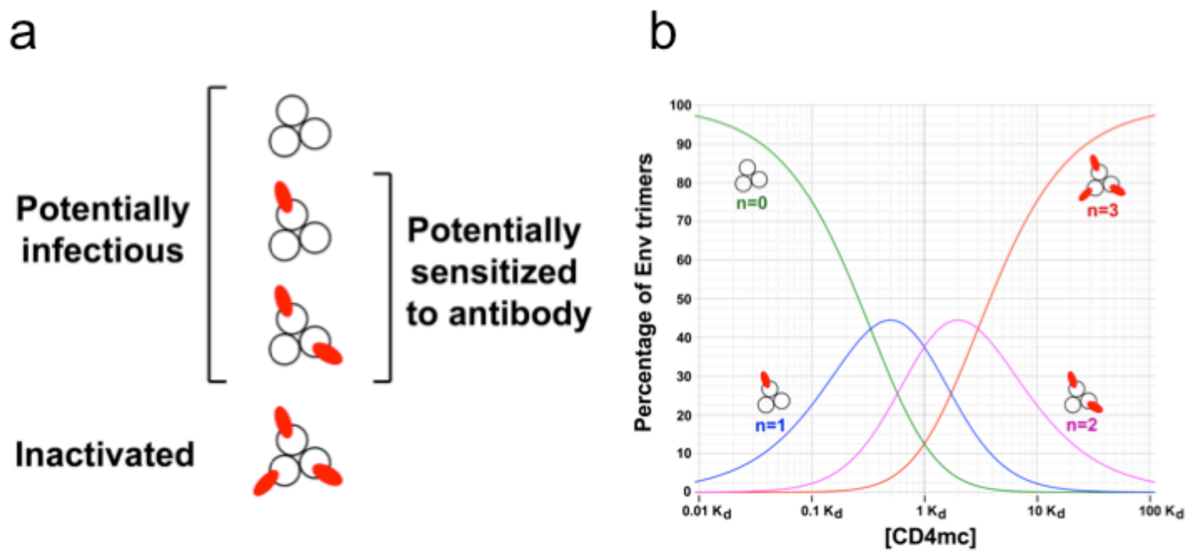

Supplementary Figure 8. Model of the interaction of the CD4mc with the HIV-1 Env trimer. (**a**) The consequences of the binding of CD4mc to the HIV-1 Env trimer depend upon the stoichiometry of binding. The three protomers of the HIV-1 Env trimer are represented as circles, and the bound CD4mc molecules are represented as red ovals. Env trimers with 0, 1 or 2 CD4mc bound are potentially able to mediate virus infection, whereas trimers with 3 CD4mc bound are not functional (Madani N, Princiotto AM, Zhao C, Jahanbakhshsefidi F, Mertens M, Herschhorn A, Melillo B, Smith AB III and Sodroski J. 2017. Activation and inactivation of primary human immunodeficiency virus envelope glycoprotein trimers by CD4-mimetic compounds. J. Virol. 91:e01880-16). Env trimers with two CD4mc bound can potentially mediate virus infection if contact with CCR5-expressing cells occurs before the Env trimer undergoes

inactivation. Env trimers with one or two CD4mc bound are potentially sensitized to neutralization by antibodies that recognize downstream conformations of Env. In the absence of bound CD4mc, HIV-1 Envs are typically resistant to neutralization by these antibodies. (**b**) The binding of the CD4mc (red ovals) to the three protomers of the HIV-1 Env trimer was modeled using a binomial distribution, as described in the Online Methods. The percentage of Env trimers in the population with a given number, n, of CD4mc bound is shown as a function of the CD4mc concentration on a semi-log plot. The CD4mc concentration is shown relative to the dissociation constant (K_d) of the CD4mc for the particular Env trimer. In the experimental protocol used in this study, the CD4mc BNM-III-170 was added to the SHIV-C5 challenge virus at a final concentration of approximately 3-6 times the estimated K_d of the compound for the HIV-1_{C5} Env trimer. At this BNM-III-170 concentration, the vast majority of Env trimers will have at least one CD4mc bound and therefore will be subject to inactivation or sensitization to antibody neutralization. A small fraction of the viral Envs will be free of CD4mc under these experimental conditions (green curve) and thus can mediate infection while resisting antibody neutralization. The infection of the Group 3 monkeys by these viruses explains the stochastic selection from the challenge stock of the infecting SHIVs, which remain sensitive to BNM-III-170 and BNM-III-170-enhanced neutralization. As expected for antibody-resistant viruses, SHIV-C5 infection did not necessarily correlate with levels of antibodies against Env in the Group 3 monkeys.

Supplementary Figure 9

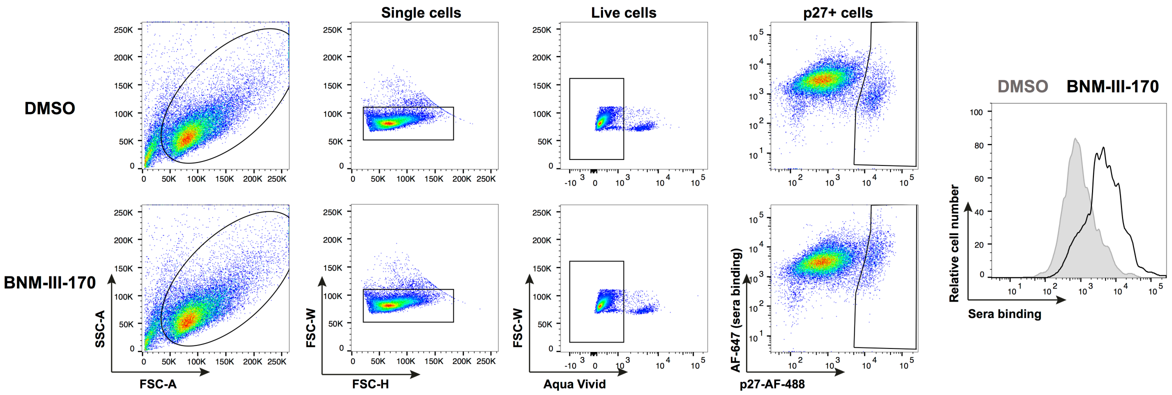

Supplementary Figure 9. Representative flow cytometry gating strategy. Primary CD4+ T cells infected with SHIV-C5 were stained with monkey sera in the presence of BNM-III-170 (50 μM) or an equivalent volume of the vehicle (DMSO). Alexa-Fluor 647 (AF-647)-conjugated goat anti-human antibody was used as a secondary antibody and Aqua Vivid as a vital dye to distinguish live and dead cells. Infected cells were identified by intracellular staining of the p27 capsid protein, using the Cytofix/Cytoperm Fixation/Permeabilization kit and the Alexa-Fluor 488 (AF-488)-conjugated p27 antibody. Mean Fluorescence Intensity (MFI) of AF-647 staining was measured on single/live/p27+ cells.

Supplementary Table 1. Study design.

Group	Monkey	Mamu A*01	Immunogen	Antibody treatment[a]	Challenge[b]
1	6704	+	Human serum albumin	None	SHIV-C5 + BNM-III-170
	6705	+		None	
	6721	–		None	
	6710	–		None	
	6711	–		None	
	6712	–		None	
2	6105	+	HIV-1_{CH505} gp120	M2850 (1)	SHIV-C5 + DMSO
	6120	–		M2850 (1)	
	6160	+		Basiliximab (1)	
	6193	+		Basiliximab (0.2)	
	6200	–		CH65 (1)	
	6203	–		CH65 (1)	
	6204	–		Basiliximab (1)	
	6214	–		Basiliximab (0.04)	
3	6104	+	HIV-1_{CH505} gp120	Basiliximab (0.04)	SHIV-C5 + BNM-III-170
	6107	+		CH65 (1)	
	6117	–		Basiliximab (1)	
	6125	–		M2850 (1)	
	6132	–		M2850 (0.2)	
	6202	–		M2850 (0.2)	
	6209	–		Basiliximab (0.2)	
	6210	+		M2850 (0.2)	

[a]Monkeys in Groups 2 and 3 were immunized as part of a previous study (CHAVI-ID NHP #109). In NHP #109, the monkeys were immunized with monomeric HIV-1_{CH505} gp120 six times. Five days after the first four immunizations, the monkeys received an intravenous infusion of the indicated antibody at the dose (in mg/kg body weight) shown in parentheses. Subsequent immunizations of the monkeys in Groups 2 and 3 with HIV-1_{CH505} gp120 were not accompanied by antibody treatment (see Supplementary Figure 1 and Online Methods).

[b]For Challenges 1-5, 3.5 animal infectious doses (AID_{50}) of SHIV-C5 were mixed with either BNM-III-170 (final concentration 300 μM) or an equivalent volume of DMSO. Within 30 minutes of preparation, the virus-compound mixtures were applied intrarectally in an atraumatic manner.

SHIV-C5 Challenges 1, 2 and 3 were each conducted 2-4 weeks after boosting the monkeys with the appropriate immunogen, either gp120 or human serum albumin. The monkeys were not immunized after Challenge 3. Thus, SHIV-C5 Challenges 4, 5 and 6 were conducted on monkeys that had last been immunized 17, 24 and 33 weeks earlier, respectively.

		HIV-1$_{AD8}$ (Clade B)		HIV-1$_{JR-FL}$ (Clade B)			HIV-1$_{C5}$ (Clade C)			A-MLV	
Monkey	BNM-III-170 (μM):	0	20	0	15	50	0	15	50	0	80
Group 1	6704	<40	<40	ND	ND	ND	ND	ND	ND	<40	<40
	6705	<40	<40	ND	ND	ND	ND	ND	ND	<40	<40
	6721	<40	<40	ND	ND	ND	ND	ND	ND	<40	<40
	6710	<40	<40	ND	ND	ND	ND	ND	ND	<40	<40
	6711	<40	<40	ND	ND	ND	ND	ND	ND	<40	<40
	6712	<40	<40	ND	ND	ND	ND	ND	ND	<40	<40
Group 2	6105	<40	**640**	<80	**640**	**>1280**	<80	**640**	**>1280**	<80	<80
	6120	<40	**640**	<80	**640**	**1280**	<80	**640**	**>1280**	<80	<80
	6160	<40	**640**	<80	<80	**>1280**	<80	**640**	**>1280**	<80	<80
	6193	<40	**640**	<80	**640**	**>1280**	80	**1280**	**>1280**	<80	80
	6200	<40	**320**	<80	**160**	**640**	<80	**1280**	**>1280**	<80	<80
	6203	<40	**640**	<80	**640**	**320-640**	<80	**640**	**>1280**	<80	<80
	6204	<40	**>640**	<80	**80**	**>1280**	<80	**1280**	**>1280**	<80	<80
	6214	<40	**640**	<80	**160**	**>1280**	<80	**640**	**>1280**	<80	<80
Group 3	6104	<40	**>640**	<80	**160**	**>1280**	<80	**640**	**>1280**	<80	<80
	6107	<40	**640**	<80	**1280**	**>1280**	<80	**1280**	**>1280**	<80	<80
	6117	<40	**>640**	<80	**1280**	**>1280**	<80	**1280**	**>1280**	<80	<80
	6125	<40	**>640**	<80	**1280**	**1280**	80	**1280**	**>1280**	<80	<80
	6132	<40	**640**	<80	**640**	**1280**	<80	**1280**	**>1280**	<80	<80
	6202	<40	**640**	<80	**160**	**320**	<80	**1280**	**>1280**	<80	<80
	6209	<40	**>640**	<80	**80**	**>1280**	<80	**1280**	**>1280**	<80	<80
	6210	<40	**640**	<80	**640**	**1280**	<80	**1280**	**>1280**	<80	<80
	17b Antibody	>30 μg/ml	0.2 μg/ml	>30 μg/ml	<0.2 μg/ml	6.5 μg/ml	>30 μg/ml	<0.2 μg/ml	<0.2 μg/ml	>30 μg/ml	>30 μg/ml

Supplementary Table 2. Neutralizing activity of the sera from gp120-immunized monkeys against recombinant HIV-1 with Envs from Clade B and Clade C HIV-1.[a]

[a]Sera collected from immunized monkeys prior to SHIV-C5 Challenge 1 were tested for the ability to neutralize HIV-1 variants in the absence and presence of subneutralizing concentrations of BNM-III-170. Recombinant luciferase-expressing HIV-1 was pseudotyped with the Envs from the indicated HIV-1 strains, with the HIV-1 phylogenetic clade shown in parentheses. A control recombinant HIV-1 pseudotyped with the envelope glycoproteins of the amphotropic murine leukemia virus (A-MLV) was studied in parallel. The recombinant viruses were incubated with the indicated concentrations of BNM-III-170 at 37°C for 30 minutes, followed by incubation at 37°C with different dilutions of the sera from the monkeys for an additional 30 minutes. In parallel experiments, the 17b antibody (over a range of 0 to 100 μg/ml) was added to the virus-BNM-III-170 mixture and incubated at 37°C for 30 minutes. The mixtures were added to Cf2Th-CD4/CCR5 target cells and, 48 hours later, luciferase activity in the cells was measured. The mean luciferase activity values derived from triplicate parallel infections were plotted relative to the values obtained in the absence of added serum/antibody. The reciprocal titer of the serum (or the concentration of 17b antibody) that resulted in 50% inhibition of virus infection compared with the level of infection in the absence of added serum/antibody is reported. Serum titers associated with sensitization of the virus by BNM-III-170 are shown in bold. ND, not determined.

Supplementary Table 3. Sequence similarity of HIV-1$_{C5}$, HIV-1$_{CH505}$ and other Clade C Envs.

Reference sequence	Accession number (Protein ID)	Subtype (Geographic origin)	% Identity, Gaps[a]	
			CH505.TF	C5 - 1245045
CH505.TF (703010505.TF)	KC247556.1 (AGG24895.1)	C (Malawi)	100%, 0	81.97%, 27
CH505 wk100 (703010505.wk100)	KC247375 (AGG24192.1)	C (Malawi)	97.52%, 5	81.13%, 22
C5 - 1245045	HQ595742 (ADR66521.1)	C (South Africa)	81.97%, 27	100%, 0
BR025	U52953 (AAB61124.1)	C (Brazil)	81.35%, 16	83.41%, 23
ETH2220	U46016 (AAB36507.1)	C (Ethiopia)	79.71%, 21	79.76%, 20
98IN012	AF286231 (AAK31033.1)	C (India)	82.34%, 33	81.31%, 22
04ZASK146	AY772699 (AAV41353.1)	C (South Africa)	81.20%, 20	80.62%, 31
ZM246F	FJ496192 (ACR53189.1)	C (Zambia)	80.58%, 15	80.53%, 36
CH0131	KC894107 (AGV38407.1)	C (Malawi)	81.67%, 41	81.68%, 36
CH0185	KC156129 (AGF30504.1)	C (South Africa)	80.99%, 31	81.80%, 26
CH0067	KC156125 (AGF30468.1)	C (South Africa)	78.66%, 21	78.69%, 30
CH0200	KC149183 (AGH69333.1)	C (Malawi)	81.21%, 18	80.14%, 25
CH0164	KC894125 (AGV38472.1)	C (South Africa)	81.26%, 15	81.74%, 30
Clade C consensus sequence	ABD57745.1	C	86.45%, 17	87.02%, 36
HXB2	K03455 (AAB50262.1)	B (USA)	73.29%, 26	73.49%, 36
JR-FL	AY669728 (AAT67500.1)	B (USA)	74.49%, 23	75.47%, 32

[a]The Env amino acid sequences of HIV-1$_{CH505.TF}$ and HIV-1$_{CH505.wk100}$, which correspond to the sequence of the gp120 immunogens, were aligned in Clustal Omega with those of HIV-1$_{C5}$ (in the challenge SHIV). Comparison was also made with Envs from reference Clade C and Clade B HIV-1 strains. The percentage of amino acid identity and gaps in each pairwise alignment are indicated.
